# Craniosynostosis: orofacial and oral health perspectives with masticatory insights

**DOI:** 10.1186/s12903-024-04540-y

**Published:** 2024-07-08

**Authors:** Yanisa Wongbanthit, Nond Rojvachiranonda, Soranun Chantarangsu, Preeya Suwanwitid, Wuttichart Kamolvisit, Thantrira Porntaveetus

**Affiliations:** 1 Center of Excellence in Genomics and Precision Dentistry, Department of Physiology, Faculty of Dentistry, Chulalongkorn University, Bangkok, 10330 Thailand; 2 Clinical Research Center, Faculty of Dentistry, Graduate Program in Geriatric and Special Patients Care, Chulalongkorn University, Bangkok, Thailand; 3 Department of Surgery, Faculty of Medicine, Chulalongkorn University, Bangkok, Thailand; 4 Princess Sirindhorn Craniofacial Center, King Chulalongkorn Memorial Hospital, Thai Red Cross Society, Bangkok, Thailand; 5 Department of Oral Pathology, Faculty of Dentistry, Chulalongkorn University, Bangkok, Thailand; 6 Department of Orthodontics, Faculty of Dentistry, Chulalongkorn University, Bangkok, Thailand; 7 Center of Excellence for Medical Genomics, Department of Pediatrics, Faculty of Medicine, Chulalongkorn University, Bangkok, Thailand; 8 Excellence Center for Genomics and Precision Medicine, King Chulalongkorn Memorial Hospital, the Thai Red Cross Society, Bangkok, Thailand

**Keywords:** Cranial suture, Dental caries, Oral hygiene, Cleft palate, Malocclusion, Openbite, Tooth eruption

## Abstract

**Background:**

Craniosynostosis (CS), premature fusion of one or more cranial sutures, leads to abnormal skull development, impacting both facial esthetics and oral function. This study aimed to evaluate the specific orofacial and oral health characteristics, including masticatory performance, in Thai patients with CS.

**Methods:**

A comparative study was conducted with Thai CS patients aged 6–17 years and a control group of healthy individuals with similar age distribution. Assessments included craniofacial morphology, oral health status, and masticatory performance. Intergroup comparisons utilized appropriate statistical tests.

**Results:**

The study included 24 CS patients with a mean age of 10.11 ± 2.98 years and 30 controls. CS patients exhibited a significantly higher prevalence of various oral conditions compared to controls: cleft palate (20.8%), anterior open bite (41.7%), anterior crossbite (54.2%), posterior crossbite (50%), combined anterior–posterior crossbite (45.8%), dental crowding in both maxilla and mandible (50% and 45.8% respectively), congenitally missing teeth (50%), supernumerary teeth (12.5%), and eruption failure (54.2%). Furthermore, CS patients exhibited significantly higher caries prevalence and susceptibility, alongside poorer oral hygiene, compared to controls. Regarding jaw relationships, CS patients exhibited a significantly higher proportion of Angle's Class III malocclusion (50%) compared to the control group, where Class I malocclusion was predominant (50%). Masticatory performance, assessed using the two-color gum mixing ability test, showed significantly higher hue variance in CS patients (0.12 ± 0.07) compared to the control group, indicating reduced chewing performance.

**Conclusion:**

This study underscores the significant orofacial and oral health challenges faced by children with CS, including a high prevalence of malocclusions, dental anomalies, elevated caries experience, and compromised masticatory function. These findings emphasize the importance of tailored interventions and comprehensive oral healthcare strategies to address the unique needs of this population and improve their overall quality of life.

**Supplementary Information:**

The online version contains supplementary material available at 10.1186/s12903-024-04540-y.

## Introduction

Craniosynostosis (CS) is a pathological condition characterized by the untimely fusion of one or more cranial sutures. The premature fusion of these sutures disrupts the natural growth patterns of the cranium, resulting in aberrant cranial morphology [1]. This deformity may lead to a spectrum of complications, including heightened intracranial pressure, developmental delays, and especially potential perturbations in cerebral and facial growth [2]. CS can be influenced by both genetic and environmental factors. Genetic variants implicated in CS have been identified in the genes encoding fibroblast growth factor receptors *(FGFRs), EFNB1*, as well as the transcription factors *TWIST* and *MSX2* [3]*.* Advanced paternal age, maternal thyroid disorder, cigarette smoking, alcohol consumption, and maternal use of antidepressant medication are among the factors implicated in the development of CS [1, 4].

The prevalence of CS in the global population is estimated to be 1 in every 2,100–2,500 live births and has been increasing [5]. CS anomalies can be observed in individuals of diverse nationalities, spanning various geographic regions, and irrespective of socioeconomic status. It can manifest as either an isolated anomaly (non-syndromic CS) or as part of a syndrome (syndromic CS) associated with other developmental anomalies.

CS patients often exhibit facial manifestations encompassing midface hypoplasia, facial asymmetry, malocclusion, and dental crowding [6]. The severity of the condition varies depending on several factors, including the quantity and location of the affected cranial sutures, along with the individual's distinctive anatomical characteristics. Craniofacial and oral issues such as class III malocclusions, supernumerary teeth, and tooth agenesis can present challenges in maintaining oral hygiene and elevate the risk of various oro-dental pathologies [7–11]. Specifically, these issues can contribute to the development of dental caries, periodontal deterioration, and oral infections. Dental plaque deposits and periodontal attachment loss in the posterior teeth are common in CS patients [12]. The altered cranial and facial structures may also affect masticatory performance. Despite various studies on CS, there is still a notable gap in our understanding of the cranio-oro-facial characteristics and masticatory performance in Thai CS patients. Therefore, the aims of this study were to investigate the craniofacial, orodental, and oral health features of these patients, creating a valuable database for CS patients. Additionally, the study sought to gain an understanding of how synostosis features relate to patients' chewing ability and overall oral health.

## Materials & methods

### Patient recruitment and ethical considerations

Thai subjects with CS enrolled at the Hospital Craniofacial Center participated in this study. The participants’ ages ranged from 6–17 years old. This study received ethical approval from the Institutional Review Board, Faculty of Medicine, Chulalongkorn University (COA no. 1348/2021, Approval date October 6, 2020) and in accordance with the 1964 Helsinki declaration and its later amendments. All participants and/or their legal guardians provided written informed consent for participation in the study and publication of their data.

The study cohort consisted of fifty-four participants (24 CS patients and 30 controls), comprising 30 females (55.6%) and 24 males (44.4%). The average age at the initial visit was 10.11 years old, with a standard deviation (SD) of 2.98 years. Patients with CS (n = 24) were classified into two groups: syndromic and nonsyndromic CS, according to clinical examination, genetic testing, imaging, and/or family history. The clinical diagnoses encompassed syndromic CS (14 patients), including Apert syndrome, Craniofrontonasal dysplasia, Pfeiffer syndrome, and Crouzon syndrome, as well as non-syndromic CS (10 patients), including coronal CS, lambdoid CS, and multiple suture CS. The details of each CS patient are shown in Table 1.

**Table 1:** Details of each patient with craniosynostosis (CS)

Patients	Clinical diagnoses	Syndromic (S) or Non-syndromic (NS)	Gender	Age at initial visit (years)
CS-1	Right coronal craniosynostosis	NS	Female	7
CS-2	Apert syndrome	S	Female	7
CS-3	Apert syndrome	S	Male	10
CS-4	Frontonasal syndrome with left coronal craniosynostosis	S	Male	6
CS-5	Apert syndrome	S	Female	15
CS-6	Craniofrontonasal dysplasia with coronal craniosynostosis	S	Female	10
CS-7	Pfeiffer syndrome	S	Male	7
CS-8	Lambdoid craniosynostosis	NS	Male	7
CS-9	Bilateral coronal craniosynostosis	NS	Female	8
CS-10	Microcephaly with bilateral coronal craniosynostosis	NS	Female	9
CS-11	Multiple sutures craniosynostosis	NS	Female	14
CS-12	Right coronal craniosynostosis	NS	Female	10
CS-13	Left coronal craniosynostosis	NS	Male	10
CS-14	Right coronal craniosynostosis	NS	Female	8
CS-15	Pfeiffer syndrome	S	Male	9
CS-16	Crouzon syndrome	S	Male	10
CS-17	Crouzon syndrome	S	Female	10
CS-18	Apert syndrome	S	Male	12
CS-19	Multiple sutures craniosynostosis	NS	Female	6
CS-20	Crouzon syndrome	S	Male	13
CS-21	Multiple sutures craniosynostosis	S	Male	6
CS-22	Multiple sutures craniosynostosis	NS	Female	8
CS-23	Pfeiffer syndrome	S	Male	17
CS-24	Craniofrontonasal dysplasia with coronal craniosynostosis	S	Female	8

### Variables collected/recorded

The subjects’ demographic data were collected from their medical records. For primary teeth, the dmft index is calculated by summing the number of decayed (d), missing (m), and filled (f) teeth after a clinical examination by a trained dentist. For permanent teeth, the DMFT index is calculated similarly by summing the decayed (D), missing (M), and filled (F) teeth. The DMFS index for permanent teeth is derived by summing the decayed (D), missing (M), and filled (F) surfaces of each tooth, while the dmfs index for primary teeth is calculated by summing the decayed (d), missing (m), and filled (f) surfaces. The Simplified Oral Hygiene Index (OHI-S) was used to record oral health status [13]. The index comprises two components: the Debris Index (DI-S) and the Calculus Index (CI-S), both scored on a scale of 0 to 3 for each of the six tooth sextants. The OHI-S score is calculated by adding the DI-S and CI-S scores and dividing by 6, resulting in a range from 0 to 6. A lower score indicates better oral hygiene. Additionally, caries risk assessment, which measured biological, protective, and clinical factors to determine high, moderate, or low risk [14], and classification of dental malocclusion based on Angle's classification, were performed. The tooth numbering system utilized followed The Federation Dentaire Internationale (FDI) Numbering System. The intra-observer reliability test was conducted by re-evaluating 10 randomly selected participants for OHI-S, DMFT/dmft, and DMFS/dmfs at a minimum interval of two weeks. The intraclass correlation coefficient demonstrated a value of 0.997 (95% CI 0.995–0.998), signifying excellent agreement.

### Masticatory performance

The two-color chewing gum mixing ability test was employed for evaluating masticatory performance. The chewing gum used in this study was Hubba-Bubba Tape Gum (WM. Wrigley Jr. Company, Chicago, USA). Each specimen was prepared from ‘azure’ (sour berry flavor) and ‘pink’ (fancy fruit flavor) gum. Strips measuring 30 mm in length were precisely cut from both colored gums and manually affixed together [15]. Each participant was instructed to chew a piece of fresh gum for 20 cycles. Subsequently, the chewed gum was carefully placed in a labeled plastic bag and compressed into 1-mm thick wafers. Both sides of the wafers were scanned using a flatbed scanner (Brother MFC-J2330DW Multifunction Inkjet Printer and Scanner) on the day it was chewed, and the resulting images were analyzed using the ViewGum© software. This software quantified the standard deviation of hue or the variance of hue histogram, which has been established as a reliable and optimal method for evaluating masticatory performance [16]. The intra-examiner reliability of the masticatory performance test was determined to be 0.996 (95% CI 0.982–0.999), indicating a high level of agreement.

### Statistical analysis

The statistical analyses were performed using IBM SPSS for Windows version 22.0 (IBM; Armonk, NY, USA). The significance level was set at *p*-value < 0.05. The data were evaluated for a normal distribution using the Shapiro–Wilk test. Differences in proportion between the case and control groups were analyzed using the Chi-square test or Fisher's exact test. Differences in the quantitative variables between the case and control groups were accessed using the Mann–Whitney U test or independent* t*-test.

## Results

**Fig. 1:**
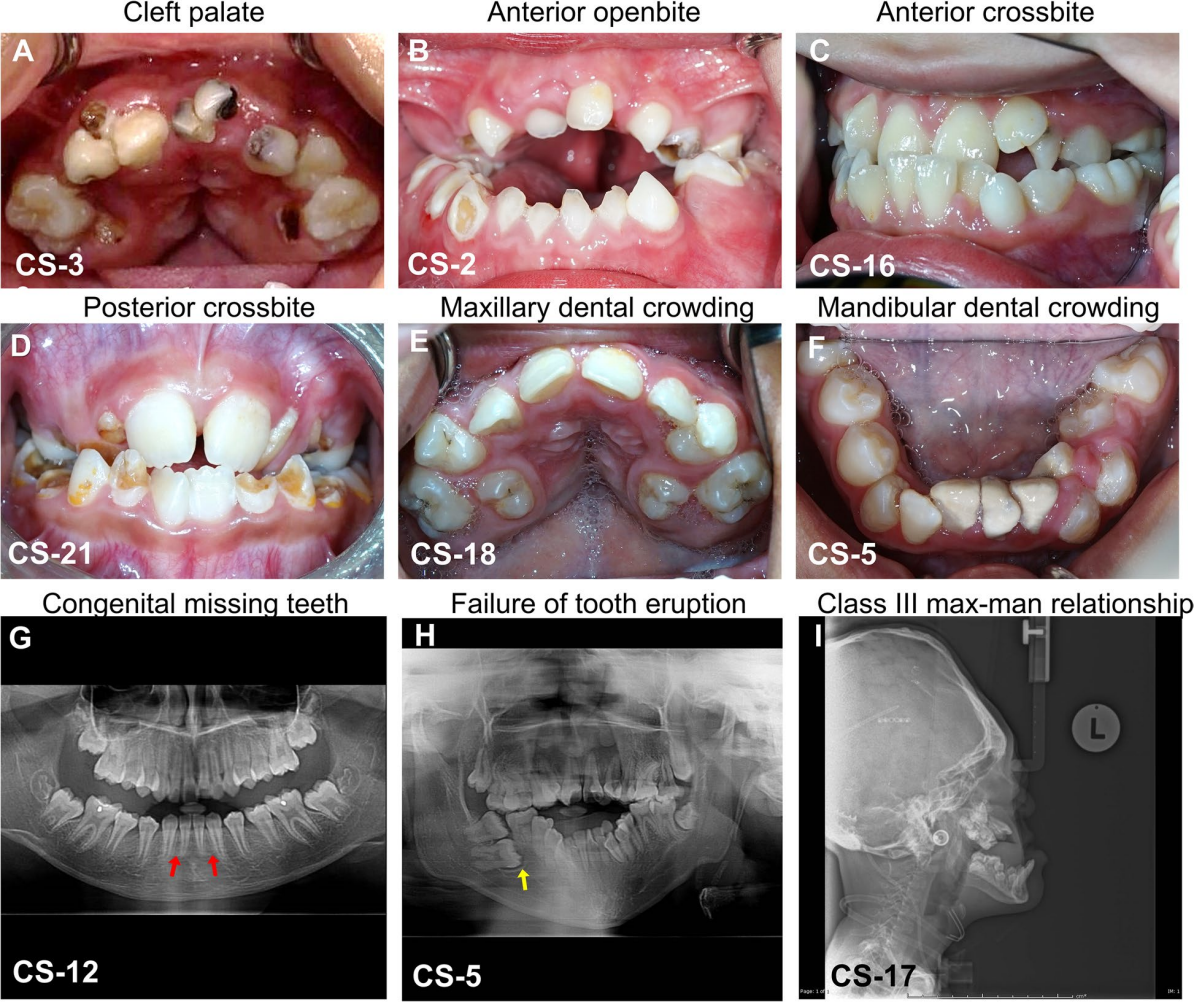
Intraoral features and radiographic images of craniosynostosis (CS) patients.** A** A 10-year-old boy diagnosed with Apert syndrome (CS-3) showed cleft palate. **B** A 7-year-old girl with Apert syndrome (CS-2) manifested anterior openbite due to underdeveloped maxilla.** C** A 10-year-old boy with Crouzon syndrome (CS-16) demonstrated anterior crossbite. **D** A 6-year-old boy with multiple sutures CS (CS-21) exhibited posterior crossbite.** E** A 12**-**year-old boy with Apert syndrome (CS-18) had maxillary dental crowding**. F** A 15-year-old girl with Apert syndrome (CS-5) manifested mandibular dental crowding.** G** Panoramic film of A 9-year-old girl with Apert syndrome (CS-2) exhibited congenital missing lower lateral incisor teeth (arrows). **H** Panoramic film of a 15-year-old girl with Apert syndrome (CS-5) showed failure of tooth eruption (arrow).** I** Lateral cephalogram of a 10-year-old girl with Crouzon syndrome (CS-17) demonstrated Class III maxillomandibular relationship

The intraoral examinations conducted on patients with CS revealed that 20.8% (5/24) presented with a cleft palate (Fig. 1A), 41.7% (10/24) with an anterior openbite (Fig. 1B), 54.2% (13/24) with an anterior crossbite (Fig. 1C), 50% (12/24) with a posterior crossbite (Fig. 1D), and 45.8% (11/24) with both anterior–posterior crossbite. Tooth crowding in the maxilla (Fig. 1E) and in the mandible (Fig. 1F) was evident in 50% (12/24) and 45.8% (11/24) of the patients, respectively (Table 1). Congenital missing teeth (Fig. 1G) were detected in 50% (12/24), supernumerary teeth in 12.5% (3/24), and a failure of tooth eruption (Fig. 1H) in 54.2% (13/24) of the CS patients. Conversely, the control group of healthy individuals did not exhibit any of the aforementioned intraoral findings.

**Fig. 2:**
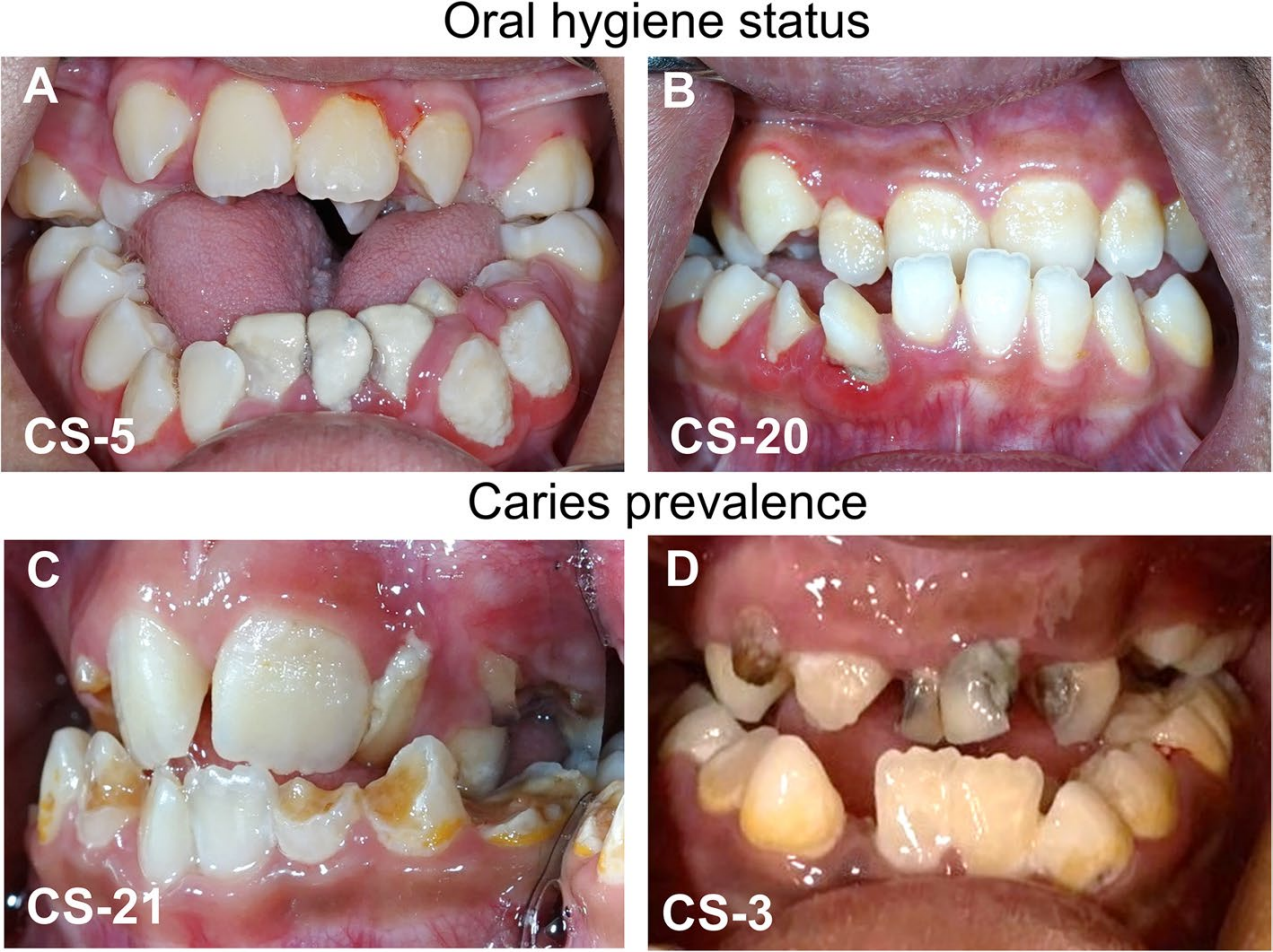
Oral health status of CS patients.** A** A 15-year-old girl diagnosed with Apert syndrome (CS-5) exhibited poor oral hygiene status.** B** A 13-year-old boy with Crouzon syndrome (CS-20) had fair oral hygiene status. **C** A 6-year-old boy with multiple sutures CS (CS-21) showed high dental caries prevalence.** D** A 10-year-old boy with Apert syndrome (CS-3) had high dental caries prevalence

The OHI-S score of the CS patients was 1.92 ± 0.84 (Table 2 and Fig. 2A, B), while the OHI-S score of the controls was 1.32 ± 0.67. The OHI-S score was statistically higher in the CS patients compared to the control group, suggesting poorer oral hygiene practices among CS patients. Caries prevalence was also found to be significantly higher in the CS patient group. This was evident across various dental caries indices, including dmft, DMFT, dmfs, and DMFS (Table 2 and Fig. 2C, D). The numbers of CS patients who exhibited dmft, DMFT, dmfs, and DMFS > 0 were 75.0% (18/24), 58.3% (14/24), 75.0% (18/24), and 58.3% (14/24), respectively, while those of the controls were 23.3% (7/30), 13.3% (4/30), 23.3% (7/30), and 13.3% (4/30), respectively. A statistically significantly higher proportion of CS patients fell into the high caries prevalence category (dmft, DMFT, dmfs, and DMFS > 0) compared to controls. Detailed information on the distribution and descriptive statistics of caries prevalence were shown in Supplementary Tables S1 and S2.

**Table 2:** Characteristics of the CS patients and controls

Characteristics	Total (*N* = 54)	CS (*N* = 24)	Control (*N* = 30)	*p*-value
Age (year), mean ± SD^b^	10.11 ± 2.98	9.50 ± 2.99	10.60 ± 2.92	0.138
Sex, *N* (%)^c^				
Male	24 (44.4%)	11 (45.8%)	13 (43.3%)	0.854
Female	30 (55.6%)	13 (54.2%)	17 (56.7%)	
Cleft palate, *N* (%)^d^	5 (9.3%)	5 (20.8%)	0 (0%)	0.013*
Anterior openbite, *N* (%)^d^	10 (18.5%)	10 (41.7%)	0 (0%)	< 0.001*
Anterior crossbite, *N* (%)^c^	13 (24.1%)	13 (54.2%)	0 (0%)	< 0.001*
Posterior crossbite, *N* (%)^c^	12 (22.2%)	12 (50%)	0 (0%)	< 0.001*
Anterior–Posterior crossbite, *N* (%)^c^	11 (20.4%)	11 (45.8%)	0 (0%)	< 0.001*
Dental crowding, *N* (%)				
Maxillary arch^c^	12 (22.2%)	12 (50%)	0 (0%)	< 0.001*
Mandibular arch^d^	11 (20.4%)	11 (45.8%)	0 (0%)	< 0.001*
Congenital missing teeth, *N* (%) ^a,c^	12 (22.2%)	12 (50%)	0 (0%)	< 0.001*
Supernumerary teeth, N (%) ^a,d^	3 (5.6%)	3 (12.5%)	0 (0%)	0.082
Failure of tooth eruption, N (%)^a,c^	13 (24.1%)	13 (54.2%)	0 (0%)	< 0.001*
Oral hygiene status				
OHI-S, mean ± SD^e^	1.58 ± 0.80	1.92 ± 0.84	1.32 ± 0.67	0.005*
Caries prevalence				
dmft, *N* (%)^c^				< 0.001*
dmft = 0	29 (53.7%)	6 (25.0%)	23 (76.7%)	
dmft > 0	25 (46.3%)	18 (75.0%)	7 (23.3.%)	
DMFT, *N* (%)^c^				< 0.001*
DMFT = 0	36 (66.7%)	10 (41.7%)	26 (86.7%)	
DMFT > 0	18 (33.3%)	14 (58.3%)	4 (13.3%)	
dmfs, *N* (%)^c^				< 0.001*
dmfs = 0	29 (53.7%)	6 (25.0%)	23 (76.7%)	
dmfs > 0	25 (46.3%)	18 (75.0%)	7 (23.3%)	
DMFS, *N* (%)^c^				< 0.001*
DMFS = 0	36 (66.7%)	10 (41.7%)	26 (86.7%)	
DMFS > 0	18 (33.3%)	14 (58.3)	4 (13.3%)	
Caries risk assessment, *N* (%)^c^				
High risk	15 (27.8%)	12 (50%)	3 (10%)	< 0.001*
Moderate risk	16 (29.6%)	10 (41.7%)	6 (20%)	
Low risk	23 (42.6%)	2 (8.3%)	21 (70%)	
Maxillomandibular dental relationship, N (%)^c^				
Angle’s Class I	39 (72.2%)	12 (50%)	27 (90%)	< 0.001*
Angle’s Class II	2 (3.7%)	0 (0%)	2 (6.7%)	
Angle’s Class III	13 (24.1%)	12 (50%)	1 (3.3%)	

*dmft* decayed, missing, and filled primary teeth, *DMFT* decayed, missing, and filled permanent teeth, *dmfs* decayed, missing, and filled surface in primary teeth, *DMFS* decayed, missing, and filled surface in permanent teeth

^a^The data was derived from panoramic radiographs

^b^Differences between case and control groups, analysed by the Mann–Whitney U test

^c^Differences between case and control groups, analysed by the Chi-square test

^d^Differences between case and control groups, analysed by the Fisher’s exact test

^e^Differences between case and control groups, analysed by the independent *t*-test

^*^Significant difference (*p* < 0.05)

Regarding caries risk assessment, 50% (12/24) of the CS cases exhibited a high caries risk, 41.7% (10/24) demonstrated a moderate caries risk, and 8.3% (2/24) had a low risk. Conversely, a high caries risk was found in 10% (3/30), a moderate risk in 20% (6/30), and a low risk in 70% (21/30) in the controls (Table 2). These findings underscore the high-moderate caries risk in the CS cases, which is higher compared to the control group (*p* < 0.001).

Considering the maxilla-mandibular dentition relationship, 50% (12/24) of the CS cases demonstrated Angle's Class I, while the remaining 50% displayed Angle's Class III (Fig. 1I, Table 2). Notably, no CS cases presented Angle's Class II. In contrast, the control group showed a different distribution, with 90% (27/30) exhibiting Angle's Class I, 6.7% (2/30) having Angle's Class II, and 3.3% (1/30) with Angle's Class III. These findings emphasise the notable difference in the maxillomandibular dentition relationship between the CS patients and the healthy individuals. Furthermore, it suggests that the CS patients have a higher likelihood of experiencing a deviated maxilla-mandibular tooth relationship, particularly Class III malocclusion compared to the control group (*p* < 0.001).

Several orodental abnormalities observed in the CS patients were significantly more pronounced compared with the healthy subjects (Table 2). These defects comprised 1) cleft palate (*p* = 0.013), 2) anterior openbite (*p* < 0.001), 3) anterior crossbite (*p* < 0.001), 4) posterior crossbite (*p* < 0.001), 5) anterior–posterior crossbite (*p* < 0.001), 6) maxillary teeth crowding (*p* < 0.001), 7) mandibular teeth crowding (*p* < 0.001), 8) congenital missing teeth (*p* < 0.001), 9) failure of tooth eruption (*p* < 0.001), and 10) maxillomandibular dental relationship (*p* < 0.001).

Moreover, the orodental problems were also significantly higher in the CS patients, compared with the controls. These consisted of 1) OHI-S (*p* = 0.005), 2) dmft (*p* < 0.001), 3) DMFT (*p* < 0.001), 4) dmfs (*p* < 0.001), 5) DMFS (*p* < 0.001), and 6) caries risk assessment (*p* < 0.001), However, age, sex, and the presence of supernumerary teeth were not significantly different between the CS cases and the controls. The characteristics of the CS patients and the controls are summarized in Table 2.

**Fig. 3:**
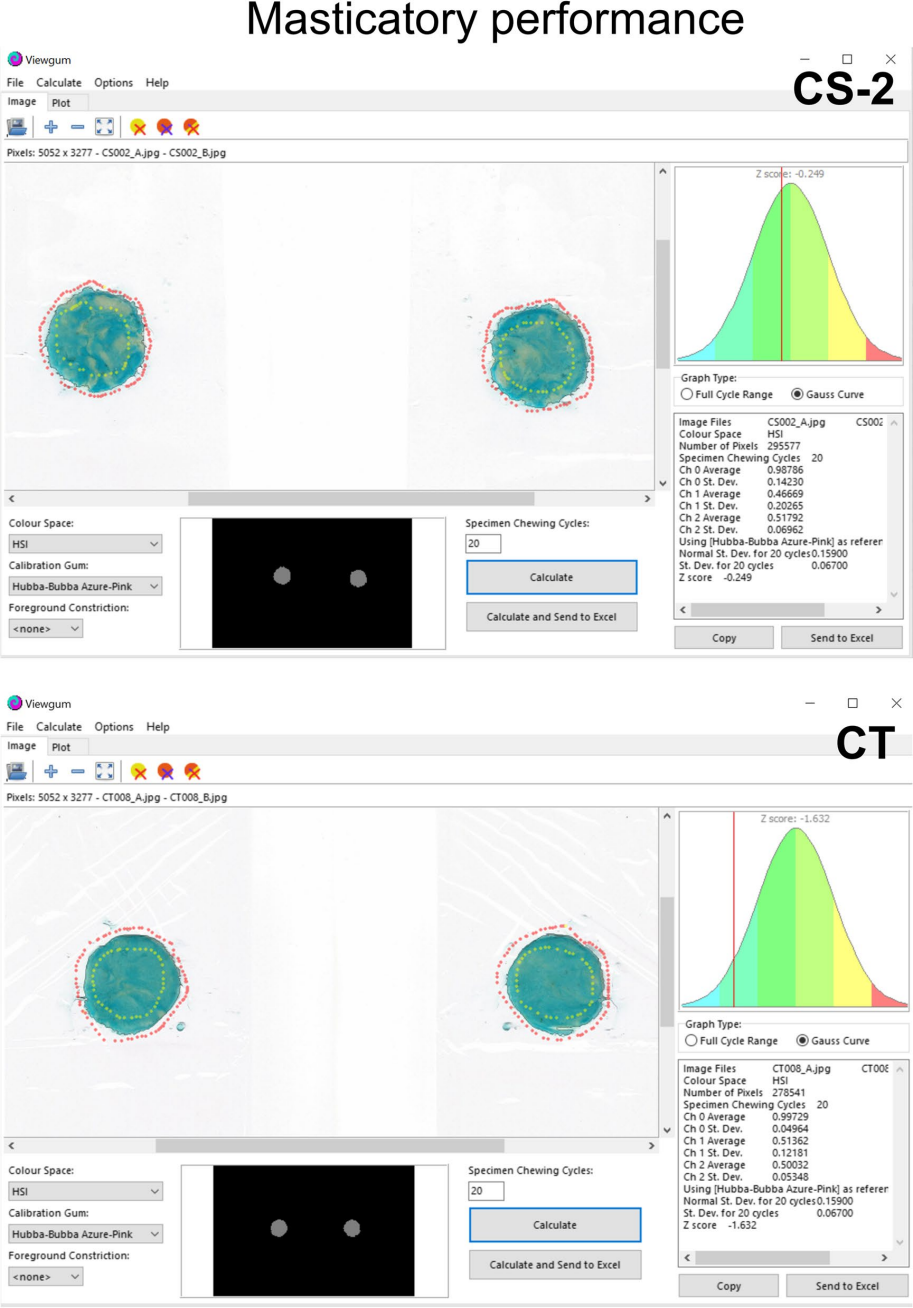
The masticatory performance of CS patients using the ViewGum© software. The software presented two scanned images of a piece of chewing gum that had undergone 20 chewing cycles, providing views of both sides of the flattened gum. The images were annotated and depicted with distinct yellow dots, while red dots were employed to indicate the background. Subsequently, the software delineated the gum area and displayed a compact thumbnail image below the primary visuals to visually represent this segmented region. CT, control

Masticatory performance was assessed using the two-color chewing gum mixing ability test, with the score of the test representing the variance of hue (VOH). The mean score in the CS patients was 0.12 ± 0.07 (Table 3), while the mean score in the control group was 0.06 ± 0.04. The comparison between the patient and control groups revealed a significant difference in masticatory performance (Mann–Whitney U test* p* < 0.001, Fig. 3). The VOH in the CS patient group was higher than in the control group.

**Table 3:** Assessment of masticatory performance in CS patients and controls

Patients	Variance of Hue	Controls	Variance of Hue
**CS-1**	0.04	**CT-1**	0.06
**CS-2**	0.14	**CT-2**	0.05
**CS-3**	0.19	**CT-3**	0.05
**CS-4**	0.18	**CT-4**	0.18
**CS-5**	0.15	**CT-5**	0.05
**CS-6**	0.09	**CT-6**	0.04
**CS-7**	0.32	**CT-7**	0.04
**CS-8**	0.13	**CT-8**	0.07
**CS-9**	0.05	**CT-9**	0.05
**CS-10**	0.06	**CT-10**	0.07
**CS-11**	0.07	**CT-11**	0.06
**CS-12**	0.12	**CT-12**	0.06
**CS-13**	0.05	**CT-13**	0.02
**CS-14**	0.07	**CT-14**	0.10
**CS-15**	0.23	**CT-15**	0.07
**CS-16**	0.14	**CT-16**	0.04
**CS-17**	0.21	**CT-17**	0.17
**CS-18**	0.17	**CT-18**	0.04
**CS-19**	0.07	**CT-19**	0.04
**CS-20**	0.13	**CT-20**	0.05
**CS-21**	0.06	**CT-21**	0.04
**CS-22**	0.03	**CT-22**	0.05
**CS-23**	0.03	**CT-23**	0.03
**CS-24**	0.08	**CT-24**	0.04
		**CT-25**	0.04
		**CT-26**	0.04
		**CT-27**	0.03
		**CT-28**	0.02
		**CT-29**	0.04
		**CT-30**	0.03
**Mean ± SD**	**0.12 ± 0.07**		**0.06 ± 0.04**

## Discussion

This study demonstrated distinctive orodental features and variations in oral health status and masticatory performance in Thai individuals with craniosynostosis. Those included cleft palate, anterior openbite, anteroposterior crossbite, maxillary-mandibular teeth crowding, congenital missing teeth, failure of tooth eruption, poor oral hygiene status, high prevalence of dental caries, elevated caries risk, and Class III malocclusion. The untimely fusion of the coronal, sagittal, and metopic sutures, combined with synostosis of the cranial base, contributes to the manifestation of sagittal and transverse maxillary underdevelopment [17]. Intraorally, this was observed through the presence of an anterior open bite, anterior and posterior crossbite, and crowding. Additionally, a narrow and elevated palatal arch was notable [18]. The concurrence of midface hypoplasia and regular mandibular growth gives rise to a Class III malocclusion in CS patients [19]. These features were found significantly more often compared with those observed in healthy Thai children. However, the presence of supernumerary teeth was not significantly different between the CS patients and the control group. This observation suggests that the presence of supernumerary teeth in individuals with CS may vary and be attributed to complex genetic and developmental factors.

The findings of this study were consistent with the results of a prior study showing that cleft palate is a common phenotypic feature found in individuals with Apert syndrome [20]. An anterior openbite and anterior–posterior crossbite were common features observed in patients with syndromic CS, including Muenke, Crouzon, Pfieffer, and Apert syndromes [9]. Maxillary hypoplasia can lead to an imbalance between the maxilla and mandible, resulting in skeletal discrepancies and significant dental crowding [21]. In 2009, Nieminen demonstrated that congenital missing teeth were consistent findings in patients with syndromic CS [22]. The abnormal maxilla-mandibular relationship observed in CS patients in this study aligns with a previous study, which showed that alterations in the growth of the anterior and posterior cranial bases can influence the skeletal relationship between the maxilla and mandible [23].

The high prevalence of Class III malocclusions (50%), cleft palate (20.8%), and anterior open bite (41.7%) in our samples suggests that a significant proportion of these patients, who are likely to have an Index of Orthognathic Functional Treatment Need (IOFTN) score of 4 or 5, may require orthognathic surgery to address functional and aesthetic concerns [24, 25]. The sample group in this study, despite presenting with craniofacial syndromes, remains in the developmental phase of jaw and facial growth. These individuals also exhibit complex craniofacial deformities and other syndromic anomalies that necessitate a specialized, multidisciplinary approach involving craniofacial surgeons, neurosurgeons, orthodontists, and plastic surgeons. The IOFTN scoring system was not utilized in this study; however, applying this measure in future studies involving older individuals with craniofacial anomalies could provide valuable insights into the specific orthognathic needs of this population.

The OHI-S of the CS patients was significantly higher than those of healthy children and exceeded the scores reported in the 8th Thai National Oral Health Survey 2017 [26]. This finding supports the notion that the CS patients are more prone to poor oral hygiene, consistent with previous studies that found a significant difference between the CS group and control in plaque score [27]. Many individuals affected by craniofacial deformities require comprehensive dental treatment due to the presence of malocclusion, suboptimal oral hygiene, and reduced salivary flow rates, which may lead to the occurrence of dental caries and periodontal disease [28]. Múfalo et al. observed that in all syndromic CS cases, posterior teeth consistently showed elevated mean probing depths and greater loss in clinical attachment level.[[12]] Moreover, a higher prevalence of dental caries was observed in individuals with cleft lip and/or cleft palate, which correlated to previous studies [29, 30]. Dalben et al. found that, based on the high prevalence of dental anomalies in CS patients, dental practitioners should provide comprehensive care to these individuals [31]. Patients with CS frequently present with distinct oral health conditions that can potentially elevate their susceptibility to dental caries and periodontal diseases [32]. Our findings revealed several factors contributing to the increased susceptibility of CS patients to food debris accumulation. These factors include cleft palate, anterior crossbite, posterior crossbite, and dental crowding, all of which pose challenges in maintaining adequate oral hygiene. Beyond the accumulation of debris, CS patients often face additional obstacles in removing accumulated debris due to various factors, including neurological damage, reduced visual acuity, impairment of the cleaning function performed by the tongue, motor impairment for correct manual hygiene, and reduced salivary flow. These challenges further complicate their oral health management. Additionally, CS patients often exhibit dentofacial abnormalities, such as midface hypoplasia, maxillary constriction, and facial imbalance, which further elevate the risk of developing dental cavities and gingival inflammation. Masticatory performance may also be impaired by malocclusion, abnormal orofacial myofunctional status, and craniofacial deformities, correlations supported by prior studies [33–36]. However, the impact of dentofacial deformities on masticatory function is still debatable, and the evidence is not conclusive [37, 38]. These deformities can lead to reduced muscle strength, uncoordinated and asymmetrical movements, and uncoordinated function of the tongue during chewing. Additionally, reduced salivary flow, which plays a crucial role in oral lubrication and cleansing, can negatively impact chewing efficiency and overall oral health.

Several methods can be utilized to evaluate masticatory performance with the comminution test being considered the gold standard [39]. Other methods include color-changeable chewing gum [40] and the two-color chewing gum mixing ability test. To assess masticatory performance, this study employed the two-color chewing gum mixing ability test due to its established reliability, validity, and feasibility in previous research [15, 16]. The protocol was based on the foundational work by Halazonetis et al*.*[41] Analysis involved VOH as a quantitative parameter for evaluating color mixing within images of the chewed gum. Higher VOH values indicate inadequate mixing and, consequently, reduced masticatory performance, while lower values signify a more homogenous blend of colors and better chewing performance. [16] While the comminution test remains a gold standard, it presented significant challenges for our study population, which included individuals with severe syndromic conditions and potential dysphagia. The two-color chewing gum mixing ability test offered several advantages in this context. First, the chewing gum does not cause particle comminution or the formation of a food bolus, making this methodology potentially safer for our participants with dysphagia. Second, many children are more familiar with bubble gum compared with paraffin wax or silicone, which may lead to more accurate results, especially in children and special needs patient populations. Additionally, the Hubba-Bubba Tape Gum types used in the test are widely accessible compared with color-changeable chewing gum, which may only be available in certain countries. Despite these advantages, the Hubba-Bubba Tape Gum is not sugar-free, necessitating a water rinse after chewing to prevent dental demineralization. Furthermore, the test may not be practical for severe syndromic patients who have dysphagia conditions, a high risk of aspiration, or who are uncooperative during the procedure.

The study has certain limitations that should be acknowledged. First, the patient population was limited to individuals treated exclusively at the King Chulalongkorn Memorial Hospital, which may introduce bias and limit the generalizability of the findings to other populations treated elsewhere or with different nationalities. Second, a larger sample size would be desirable for increased statistical power and generalizability. Future multicenter studies could facilitate the recruitment of a larger and more diverse sample, potentially enabling the detection of smaller effect sizes and enhancing the generalizability of the findings. Third, data collection from the patients was challenging and time-consuming, which may introduce potential errors or limitations in the data obtained. Fourth, the assessment of masticatory performance and ability in this study might only reflect certain aspects of the patients' overall masticatory function, and additional measures or comprehensive evaluation methods could provide a more complete understanding of their masticatory abilities. To address these limitations and further enhance our knowledge about patients with this condition, future research should consider incorporating additional genetic investigations to better understand the underlying mechanisms, conducting multi-centered studies to increase the sample size and diversity of the patient population, and incorporating comprehensive evaluation methods to provide a more comprehensive assessment of masticatory functions.

To conclude, this study revealed that the CS patients exhibited various craniofacial manifestations, including midface hypoplasia, facial asymmetry, malocclusion, and dental crowding, which contributed to oral hygiene difficulties and increased the risk of dental caries, periodontal issues, and oral infections. The CS patients also presented with a higher prevalence of orodental problems, such as cleft palate, openbite, crossbite, dental crowding, congenital missing teeth, and failure of tooth eruption compared with the control group. Masticatory performance was significantly lower in the CS patients compared with the control group. The presence of craniofacial complications in the CS patients emphasizes the need to address these issues to mitigate oral health risks and ensure proper dental management.

### Supplementary Information

Supplementary Material 1.

## Data Availability

All data generated or analyzed during this study are included in this published article and its supplementary information file.

## References

[1] Johnson D, Wilkie AOM (2011). Craniosynostosis. Eur J Hum Genet.

[2] Yilmaz E, Mihci E, Nur B, Alper ÖM, Taçoy Ş (2019). Recent Advances in Craniosynostosis. Pediatr Neurol.

[3] Lenton KA, Nacamuli RP, Wan DC, Helms JA, Longaker MT (2005). Cranial suture biology. Curr Top Dev Biol.

[4] Barik M, Bajpai M, Das RR, Panda SS (2013). Study of environmental and genetic factors in children with craniosynostosis: A case-control study. J Pediatr Neurosci.

[5] Cornelissen M, Ottelander B, Rizopoulos D, van der Hulst R (2016). Mink van der Molen A, van der Horst C, Delye H, van Veelen ML, Bonsel G, Mathijssen I: Increase of prevalence of craniosynostosis. J Craniomaxillofac Surg.

[6] Taylor JA, Bartlett SP (2017). What's New in Syndromic Craniosynostosis Surgery?. Plast Reconstr Surg.

[7] Korakavi N, Prokop JW, Seaver LH (2019). Evolution of the phenotype of craniosynostosis with dental anomalies syndrome and report of IL11RA variant population frequencies in a Crouzon-like autosomal recessive syndrome. Am J Med Genet A.

[8] Leinonen S, Rice D, Leikola J, Heliövaara A (2021). Dental Age, Agenesis, and Morphology in Patients With Operated Single-Suture Craniosynostoses. Cleft Palate Craniofac J.

[9] Azoulay-Avinoam S, Bruun R, MacLaine J, Allareddy V, Resnick CM, Padwa BL (2020). An Overview of Craniosynostosis Craniofacial Syndromes for Combined Orthodontic and Surgical Management. Oral Maxillofac Surg Clin North Am.

[10] Pinto RO, Tonello C, Peixoto AP, de Jesus AS, Dos Santos-Pinto A, Raveli DB (2023). Three-Dimensional Evaluation of Dental Arches in Individuals with Syndromic Craniosynostosis. Int J Dent.

[11] Droubi L, Laflouf M, Tolibah YA, Comisi JC (2022). Apert Syndrome: Dental management considerations and objectives. J Oral Biol Craniofac Res.

[12] Múfalo PS, Kaizer Rde O, Dalben Gda S, de Almeida AL (2009). Comparison of periodontal parameters in individuals with syndromic craniosynostosis. J Appl Oral Sci.

[13] Petersen PE, Baez RJ, World Health O (2013). Oral health surveys: basic methods, 5th.

[14] American Academy of Pediatric Dentsitry (2017). Caries-risk Assessment and Management for Infants, Children, and Adolescents. Pediatr Dent.

[15] Schimmel M, Christou P, Herrmann F, Müller F (2007). A two-colour chewing gum test for masticatory efficiency: development of different assessment methods. J Oral Rehabil.

[16] Schimmel M, Christou P, Miyazaki H, Halazonetis D, Herrmann FR, Müller F (2015). A novel colourimetric technique to assess chewing function using two-coloured specimens: Validation and application. J Dent.

[17] Cohen MM, Kreiborg S (1993). Growth pattern in the Apert syndrome. Am J Med Genet.

[18] Kakutani H, Sato Y, Tsukamoto-Takakusagi Y, Saito F, Oyama A, Iida J (2017). Evaluation of the maxillofacial morphological characteristics of Apert syndrome infants. Congenit Anom.

[19] Derderian C, Seaward J (2012). Syndromic craniosynostosis. Semin Plast Surg.

[20] Willie D, Holmes G, Jabs EW, Wu M (2022). Cleft Palate in Apert Syndrome. J Dev Biol.

[21] Letra A, de Almeida AL, Kaizer R, Esper LA, Sgarbosa S, Granjeiro JM (2007). Intraoral features of Apert's syndrome. Oral Surg Oral Med Oral Pathol Oral Radiol Endod.

[22] Nieminen P (2009). Genetic basis of tooth agenesis. J Exp Zool B Mol Dev Evol.

[23] Nie X (2005). Cranial base in craniofacial development: Developmental features, influence on facial growth, anomaly, and molecular basis. Acta Odontol Scand.

[24] Ireland A, Cunningham S, Petrie A, Cobourne M, Acharya P, Sandy J, Hunt N (2014). An index of Orthognathic Functional Treatment Need (IOFTN). J Orthod.

[25] Borzabadi-Farahani A. Systematic Review and Meta-Analysis of the Index of Orthognathic Functional Treatment Need for Detecting Subjects with Great Need for Orthognathic Surgery. Cleft Palate Craniofac J 2023:10556656231216833. 10.1177/10556656231216833. Epub ahead of print. 10.1177/10556656231216833 38037271

[26] Bureau of Dental Health, Department of Health. The 8th Thai National Oral Health Survey. Nonthaburi: Sam Charoen Panich; 2017. p. 330. https://dental.anamai.moph.go.th/webupload/migrated/files/dental2/n2423_3e9aed89eb9e4e3978640d0a60b44be6_survey8th_2nd.pdf.

[27] Mustafa D, Lucas VS, Junod P, Evans R, Mason C, Roberts GJ (2001). The dental health and caries-related microflora in children with craniosynostosis. Cleft Palate Craniofac J.

[28] Vilan Xavier AC, Pinto Silva LC, Oliveira P, Villamarim Soares R, de Almeida CR (2008). A review and dental management of persons with craniosynostosis anomalies. Spec Care Dentist.

[29] Johnsen DC, Dixon M (1984). Dental caries of primary incisors in children with cleft lip and palate. Cleft Palate J.

[30] Dahllöf G, Ussisoo-Joandi R, Ideberg M, Modeer T: Caries, gingivitis, and dental abnormalities in preschool children with cleft lip and/or palate. Cleft Palate J. 1989; 26(3):233–237. 2788042

[31] Dalben Gda S (2006). das Neves LT, Gomide MR: Oral findings in patients with Apert syndrome. J Appl Oral Sci.

[32] Shin K, Moreno-Uribe LM, Allareddy V, Burton RG, Menezes AH, Fisher MD, Weber-Gasparoni K, Elangovan S (2020). Multidisciplinary care for a patient with syndromic craniosynostosis: A case report with 20 years of special care. Spec Care Dentist.

[33] Zhiyi S, Min G, Yanqi Y. The Association between Mastication, Malocclusion, and Craniofacial Morphology. Int J Dentistry Oral Sci. 2018;S1:02:002:6-11. 10.19070/2377-8075-SI02-01002.

[34] Beals SP, Joganic EF (2004). Form and function in craniofacial deformities. Semin Pediatr Neurol.

[35] Hong H, Zeng Y, Chen X, Peng C, Deng J, Zhang X, Deng L, Xie Y, Wu L (2021). Electromyographic features and efficacy of orofacial myofunctional treatment for skeletal anterior open bite in adolescents: an exploratory study. BMC Oral Health.

[36] Luo X, Huang H, Yin X, Shi B, Li J (2019). Functional stability analyses of maxillofacial skeleton bearing cleft deformities. Sci Rep.

[37] AlQahtani FA, Varma SR, Kuriadom ST, AlMaghlouth B, AlAsseri N (2024). Changes in occlusion after orthognathic surgery: a systematic review and meta-analysis. Oral Maxillofac Surg.

[38] Borzabadi-Farahani A, Olkun HK, Eslamian L, Eslamipour F: Functional needs in orthognathic patients with different sagittal skeletal discrepancies. Oral Surg Oral Med Oral Pathol Oral Radiol. 2024. 10.1016/j.oooo.2024.04.006. in press. 10.1016/j.oooo.2024.04.006 38749877

[39] Sato S, Fueki K, Sato H, Sueda S, Shiozaki T, Kato M, Ohyama T (2003). Validity and reliability of a newly developed method for evaluating masticatory function using discriminant analysis. J Oral Rehabil.

[40] Barrera LM, Buschang PH, Throckmorton GS, Roldán SI (2011). Mixed longitudinal evaluation of masticatory performance in children 6 to 17 years of age. Am J Orthod Dentofacial Orthop.

[41] Halazonetis DJ, Schimmel M, Antonarakis GS, Christou P (2013). Novel software for quantitative evaluation and graphical representation of masticatory efficiency. J Oral Rehabil.

