# Supplementary material for: Craniosynostosis: orofacial and oral health perspectives with masticatory insights
Source: BMC Oral Health. 2024 Jul 8;24:767. doi: 10.1186/s12903-024-04540-y (PMC11229286; doi:10.1186/s12903-024-04540-y)
Supplement: Supplementary file 1 — Supplementary Material 1. [file 12903_2024_4540_MOESM1_ESM.docx]

**Supplementary Data**

**Craniosynostosis: Orofacial and Oral Health Perspectives with Masticatory Insights**

Yanisa Wongbanthit^1,2^, Nond Rojvachiranonda^3,4^, Soranun Chantarangsu^5^, Preeya Suwanwitid^6^, Wuttichart Kamolvisit^7,8^, Thantrira Porntaveetus^1,2*^

^1^Center of Excellence in Genomics and Precision Dentistry, Department of Physiology, Faculty of Dentistry, Chulalongkorn University, Bangkok, Thailand

^2^Clinical Research Center, Graduate Program in Geriatric and Special Patients Care, Faculty of Dentistry, Chulalongkorn University, Bangkok, Thailand

^3^Department of Surgery, Faculty of Medicine, Chulalongkorn University, Bangkok, Thailand

^4^Princess Sirindhorn Craniofacial Center, King Chulalongkorn Memorial Hospital, Thai Red Cross Society, Bangkok, Thailand

^5^Department of Oral Pathology, Faculty of Dentistry, Chulalongkorn University, Bangkok, Thailand

^6^Department of Orthodontics, Faculty of Dentistry, Chulalongkorn University, Bangkok, Thailand

^7^Center of Excellence for Medical Genomics, Department of Pediatrics, Faculty of Medicine, Chulalongkorn University, Bangkok, Thailand

^8^Excellence Center for Genomics and Precision Medicine, King Chulalongkorn Memorial Hospital, the Thai Red Cross Society, Bangkok, Thailand

**Supplementary Table S1 Distribution and descriptive statistics of deft and DMFT regarding case (n = 24) and control (n = 30) groups.**

| **Value** | **deft, n (%)** | | **DMFT, n (%)** | |
| --- | --- | --- | --- | --- |
|  | Case | Control | Case | Control |
| 0 | 6 (25.0) | 23 (76.7) | 10 (41.7) | 26 (86.7) |
| 1 | 5 (20.8) | 1 (3.3) | 3 (12.5) | 1 (3.3) |
| 2 | 2 (8.3) |  | 1 (4.2) | 3 (10.0) |
| 3 |  | 1 (3.3) | 3 (12.5) |  |
| 4 | 1 (4.2) | 1 (3.3) | 4 (16.7) |  |
| 5 | 3 (12.5) |  |  |  |
| 6 |  | 2 (6.7) |  |  |
| 7 | 1 (4.2) | 2 (6.7) | 1 (4.2) |  |
| 9 | 1 (4.2) |  | 1 (4.2) |  |
| 10 | 2 (8.3) |  |  |  |
| 11 | 1 (4.2) |  |  |  |
| 13 | 1 (4.2) |  |  |  |
| 16 | 1 (4.2) |  |  |  |
| 17 |  |  | 1 (4.2) |  |
| Mean | 4.33 | 1.13 | 2.63 | 0.23 |
| Standard deviation | 4.80 | 2.33 | 3.91 | 0.63 |
| Median | 2.00 | 0.00 | 1.00 | 0.00 |
| Interquartile range | 8.00 | 0.00 | 4.00 | 0.00 |

**Supplementary Table S2 Distribution and descriptive statistics of defs and DMFS regarding case (n = 24) and control (n = 30) groups.**

| **Value** | **defs, n (%)** | | **DMFS, n (%)** | |
| --- | --- | --- | --- | --- |
|  | **Case** | **Control** | **Case** | **Control** |
| 0 | 6 (25) | 23 (76.7) | 10 (41.7) | 26 (86.7) |
| 1 | 2 (8.3) |  | 2 (8.3) | 1 (3.3) |
| 2 | 2 (8.3) | 1 (3.3) | 2 (8.3) | 3 (10) |
| 3 |  |  | 2 (8.3) |  |
| 4 |  |  | 3 (12.5) |  |
| 5 | 3 (12.5) |  | 2 (8.3) |  |
| 6 |  | 1 (3.3) |  |  |
| 7 | 1 (4.2) | 1 (3.3) | 1 (4.2) |  |
| 8 |  | 2 (6.7) |  |  |
| 9 | 1 (4.2) |  |  |  |
| 10 | 1 (4.2) |  |  |  |
| 13 |  | 1 (3.3) |  |  |
| 14 |  | 1 (3.3) |  |  |
| 15 |  |  | 1 (4.2) |  |
| 17 | 1 (4.2) |  |  |  |
| 19 | 1 (4.2) |  |  |  |
| 23 | 1 (4.2) |  |  |  |
| 27 | 1 (4.2) |  |  |  |
| 29 | 1 (4.2) |  |  |  |
| 31 | 1 (4.2) |  |  |  |
| 37 |  |  | 1 (4.2) |  |
| 42 | 1 (4.2) |  |  |  |
| 50 | 1 (4.2) |  |  |  |
| Mean | 11.88 | 1.93 | 3.88 | 0.23 |
| Standard deviation | 14.60 | 4.03 | 7.83 | 0.63 |
| Median | 5.00 | 0.00 | 1.50 | 0.00 |
| Interquartile range | 22.00 | 1.00 | 4.00 | 0.00 |
